# Draft genomes of seven bacterial isolates from the snow-phyllosphere interface

**DOI:** 10.1128/mra.00581-26

**Published:** 2026-10-06

**Authors:** Philip Arevalo, Lesly Bermejo, Camille Carnish, Mona Jin, Chiara Quadrani, Hannah Russak, Ronald Rauch, Elanora Smith

**Affiliations:** 1 Department of Biology, Wesleyan University 5468, Middletown, Connecticut, USA; 2 College of Integrative Sciences, Wesleyan University 5468, Middletown, Connecticut, USA; USDA-ARS San Joaquin Valley Agricultural Sciences Center, Parlier, California, USA

**Keywords:** phyllosphere-inhabiting microbes, snow-inhabiting microbes

## Abstract

The interface between snow and the phyllosphere is an ephemeral environment that presents unique opportunities for microbial life. Draft genomes of seven isolates collected snow in contact with a lawn phyllosphere in Middletown, CT, are presented here, and include representatives from the genera *Rhodococcus*, *Sphingomonas*, *Pseudomonas*, *Mucilaginibacter*, *Herbiconiux*, and *Microbacterium*.

## ANNOUNCEMENT

Phyllospheres harbor microbes capable of degrading an array of carbohydrates (1). Snow cover introduces several adaptive challenges for phyllosphere microbes, including potential nutrient stress stemming from reduced plant photosynthetic rates (2). To investigate potential adaptations of microbes inhabiting the phyllosphere under snow cover, we sampled snow that had accumulated on a mixed-species, grass-dominated lawn at Wesleyan University (Middletown, CT, USA, 41.5529635° N, 72.6560295° W) with the goal of isolating heterotrophic bacteria adapted to growth in oligotrophic environments. Twelve days after a significant winter storm, 50 mL of snow was collected aseptically from the edge of a partially melted snowbank, where the snow was in contact with the lawn surface. Snow was allowed to melt at room temperature for 45 min and diluted to 10% and 1% final concentration with filter-sterilized deionized water. Each dilution (100 μL) was plated onto Reasoner’s 2 Agar (R2A, BD Difco), and morphologically distinct colonies were selected for isolation after 1 week of incubation at room temperature. All isolates were restreaked onto R2A and allowed to grow at 28°C for 48 h before storage at 4°C. Isolates were Gram-stained and examined by light microscopy at 1,000× magnification. Fungal isolates were excluded based on cell size and the observation of budding and pseudohyphae, leaving seven bacterial isolates for further sequencing.

For genomic DNA extraction, individual colonies were picked and grown overnight in 5 mL of nutrient broth (BD Dilfco) at 28°C with shaking at 225 RPM. Cells were pelleted by centrifugation, and cell pellets were resuspended in 400 μL of DNA/RNA Shield (Zymo). DNA was extracted from resuspended pellets using the ZymoBIOMICS DNA Miniprep Kit (Zymo) with the manufacturer’s standard protocol. DNA concentration was measured using the Qubit dsDNA Broad Range assay (Invitrogen).

Whole-genome sequencing and library preparation were performed by SeqCenter (Pittsburgh, PA, USA). DNA libraries were constructed using the Illumina DNA Prep Kit with custom dual indices and sequenced on an Illumina NovaSeq X Plus sequencer to produce paired-end 151 bp reads. Demultiplexing, quality control, and adapter trimming were performed with bcl-convert v4.2.4, and reads were imported into the KBase platform (3–5) for assembly and analysis using default parameters for all steps. Reads were trimmed with Trimmomatic v0.39 (6), and the remaining paired reads were assembled with SPAdes v3.15.3 (7). Genome completeness and contamination were assessed with CheckM v1.0.18 (8), and taxonomy was assigned using GTDB-Tk v2.3.2 (9). We annotated the assemblies using DRAM v0.1.2 (10) and identified putative glycoside hydrolase (GH), polysaccharide lyase (PL), and auxiliary activity (AA) genes using an HMMER v3.3.2 (11) search of carbohydrate-active enzymes in dbCAN2 (12).

Draft genomes ranged in size from 3,725,448 to 5,690,849 bp and had GC contents between 40.1% and 70.1% (Table 1). Isolate genomes contained between 13 and 49 GH gene families, 0 and 5 PL gene families, and 3–6 AA gene families (Table 1). Five of the seven isolates remained unassigned at the species level (Table 1). These results suggest that the snow-phyllosphere interface may be rich in novel, cultivable species with significant carbohydrate-degrading potential.

**TABLE 1:** Assembly and annotation statistics

Strain	320_LB	320_CC	320_RR	320_HR	320_ES	320_PA	320_CQ
Taxonomic assignment	*Herbiconiux* sp.	*Sphingomonas* sp.	*Sphingomonas* sp.	*Mucilaginibacter* sp.	*Rhodococcus* sp.	*Microbacterium testaceum*	*Pseudomonas lutea*
Genome size (bp)	3,725,448	4,338,477	4,338,832	4,560,564	4,873,107	4,123,647	5,690,849
Contigs	10	10	12	8	12	15	50
N50 (bp)	737,172	955,900	955,900	1,117,169	831,121	517,988	207,470
GC content (%)	70.771	65.081	65.078	44.079	66.219	70.072	60.101
Read count	10,370,136	9,933,396	7,863,834	13,346,078	9,642,622	8,247,294	8,074,560
Protein-coding genes	3,412	3,972	3,971	4,093	4,522	3,266	4,991
Coverage	384.5×	292.1×	258.9×	376.5×	261.5×	282.9×	203.8×
Completeness (CheckM) (%)	99.2	99.2	99.2	98.1	97.7	99.5	99.7
Contamination (CheckM) (%)	1.01	1.16	1.16	0.71	1.32	0.0	0.54
Closest reference (GTDB-Tk)	*Herbiconiux solani* (GCF_001571005.1)	No closest reference reported	No closest reference reported	*Mucilaginibacter terrenus* (GCF_003432065.1)	*Rhodococcus trifolii* (GCF_014635345.1)	*Microbacterium testaceum_A* (GCF_900104345.1)	*Pseudomonas_E lutea* (GCA_000759445.1)
Closest reference ANI (GTDB-Tk) (%)	89.98	No closest reference reported	No closest reference reported	93.58	85.34	98.72	98.04
PL families	0	5	5	1	0	1	2
GH families	25	43	43	49	15	26	13
AA families	3	6	6	5	6	4	6
BioSample	SAMN59720156	SAMN59345545	SAMN59349852	SAMN59349849	SAMN59349851	SAMN59349850	SAMN59349848
Reads accession number	SRR39431792	SRR39431793	SRR39431794	SRR39431797	SRR39431795	SRR39431796	SRR39431798
Assembly accession number	JCAWAD000000000	JCAWAE000000000	JCAWAF000000000	JCAWAI000000000	JCAWAG000000000	JCAWAH000000000	JCAWAJ000000000

## Data Availability

All reads and assemblies for this project have been deposited at NCBI under BioProject PRJNA1463304 (Table 1). Reads were deposited to SRA under accession numbers SRR39431792, SRR39431793, SRR39431794, SRR39431797, SRR39431795, SRR39431796, and SRR39431798. Assemblies were deposited to GenBank under accession numbers JCAWAD000000000, JCAWAE000000000, JCAWAF000000000, JCAWAI000000000, JCAWAG000000000, JCAWAH000000000, and JCAWAJ000000000.
